# A Case of Facial Palsy Consequent upon Otitis

**Published:** 1849-12

**Authors:** W. H. Cobb

**Affiliations:** Louisville, Marine Hospital


Art. II.—*A Case of Facial Palsy consequent upon Otitis.* By W. H. Cobb, M. D., of the Louisville, Marine Hospital.

Thomas Allen, 31 years of age, a native of Ireland, entered the Louisville Marine Hospital, laboring under symptoms of irritability of the bladder. The patient is a man of small stature, weak and feeble frame, and at present in very bad health, though he says that until the last four or five months he has "scarcely known a day of sickness." In addition to his vesical complaint, he stated that he was troubled with a discharge of matter from his left ear, but as it was unattended with pain or suffering, no particular attention was paid to his account.

The discharge from the ear commenced about five months ago, and he attributed the attack to his having been exposed, while asleep upon the deck of a steamboat, to the damp atmosphere of one of our spring nights. Complete loss of hearing followed.

Under the treatment adopted the vesical irritability was almost entirely removed, and his general condition materially improved.

Oct. 7th. The affected ear was attacked with acute inflammation, owing most probably to exposure during the scrubbing of the ward. The usual antiphlogistic remedies were employed, and some relief to the pain, which was of the most agonizing character, was afforded. This treatment was continued for about a week with some amelioration of the suffering.

Oct. 14th. While passing through the ward, the patient called my attention to the fact of four or five small bones, or pieces of bone, as he expressed himself, having passed from his ear, and stated that he had saved one of them for me to see. This bone I found upon examination to be the malleus, and I concluded from his description of them, that the others were the remaining bones of the tympanum. The pain, as before mentioned, was of the most excruciating character, shooting through from one temple to the other. Upon closing his mouth and nostrils and blowing, air readily passed through the external ear.

Oct. 20th. The patient noticed for the first time, that the whole of the left side of the face began to swell and be painful to the touch, and soon afterwards he perceived that he had entirely lost the control of the facial muscles. He was first made aware of the paralysis while eating his supper, when he discovered that he was unable to drink his tea as it immediately ran out of the left angle of the mouth, notwithstanding his utmost endeavors to the coutrary. The appearance he presented when I first saw him, was at once ludicrous and distressing; the expression of the left side of the face was entirely gone, his mouth and the tip of his nose awry, and the eyelids wide open and incapable of closure. Upon endeavoring to contract his forehead, the right side only was wrinkled, while the left was perfectly smooth and in repose. The eye, as before mentioned, was bare and suffused with tears, which ran down over the cheek, giving rise to painful excoriations. He presented while asleep the curious phenomenon of a person sleeping with "one eye open." The eye itself was perfectly healthy with the exception of a slight degree of vascularity consequent upon its continued exposure to the rays of light and the floating particles of dust. When told to snuff in air, the left side of the nose, in consequence of the paralysis of the muscles, collapsed, and no air entered. If he attempted to blow forcibly the left cheek was distended and the air passed out of the right side of the mouth, in spite of his efforts at closure.

Combined with the palsy was an incomplete degree of anesthesia. When I pinched his cheek, he declared that he knew that something was in contact with it, but that it gave him no pain; when I laid my finger upon the affected side of the tongue he had no perception of its presence, but when any attempt was made to touch the cornea, the sudden movement of the eyeball indicated that sensation was perfect in that organ. The temporal and masseter muscles, which are supplied with motion by the motor branch of the fifth pair, were unaffected.

In reviewing the symptoms of this case, it will be seen that he had entirely lost his hearing at a period prior to his entrance into the hospital; this was probably owing to the destruction, by ulceration, of the tympanum, or some other equally important structure. The inflammation, kindled during his stay at the hospital, undoubtedly affected the bony structures of the petrous pyramid, and caries of the canal, through which the portio dura is conducted in its passage to the face, ensued. What was the cause of the paralysis? It was most probably owing, I conceive, to one of two causes. 1st. Ulceration may have attacked the substance of the nerve and caused a complete severance of its fibrillæ: Or, 2nd, a portion of carious bone, or an abscess, by its pressure upon the nervous filaments, may have cut off the communication existing between the brain and the facial muscles

I shall not attempt to offer any explanation of the anesthesia, but will merely say that no symptoms of cerebral disease were present. The treatment of the paralysis consisted in the application of leeches in front and behind the ear, and revulsion by means of a series of blisters over the pes anserinus.

At the time of making this report (Nov. 10th) the patient has regained but a very slight degree of the natural motion and sensation of the affected muscles.

